# Choroidal ischemia as one cardinal sign in giant cell arteritis

**DOI:** 10.1186/s40942-022-00422-z

**Published:** 2022-09-24

**Authors:** Antonio M. B. Casella, Ahmad M. Mansour, Souza EC, Rodrigo B. do Prado, Rodrigo Meirelles, Keye Wong, Salma Yassine, Mário Luiz R. Monteiro

**Affiliations:** 1 Department of Surgery, Health Sciences Center, Londrina State University, 60 Robert Koch Av 86038, Londrina, Paraná Brazil; 2 Department of Ophthalmology, American University of Beirut, Beirut, Lebanon; 3 Division of Ophthalmology, University of São Paulo, São Paulo, Brazil; 4 Piracicaba Ophthalmological Institute, Piracicaba, São Paulo Brazil; 5 Retina Associates of Sarasota, Sarasota, FL USA; 6 Department of Ophthalmology, University of Minnesota, Minneapolis, Minnesota USA

**Keywords:** Giant cell arteritis, Choroidal hypoperfusion, Paracentral acute middle maculopathy, Fluorescein angiography, OCTA, Arteritic anterior ischemic optic neuropathy, Cotton-wool spots

## Abstract

**Purpose:**

To describe chorioretinal signs in a case series of Giant Cell Arteritis (GCA).

**Methods:**

This is a multicenter retrospective observational case series with GCA that presented with a headache and an abrupt, unilateral loss in vision. Workup included temporal artery biopsies, intravenous fluorescein angiography, optical coherence tomography (OCT), optical coherence tomography angiography (OCTA), blood levels of erythrocyte sedimentation rate (ESR), and C-reactive protein (CRP).

**Results:**

There are a total of 8 GCA instances presented. Average age was 74.5. (Range 68–83 years). The patients reported that one eye's visual loss had suddenly started, along with a fresh headache and other systemic symptoms. Eight patients exhibited choroidal ischemia, five paracentral acute middle maculopathy (PAMM) lesions, five cotton wool spots, four anterior ischemic optic neuropathy, and one central retinal arterial occlusion at the time of presentation. The average ESR at presentation was 68 mm/hr (range 4–110), and 4/6 individuals had a significant increase. The mean CRP level was 6.2 mg/dL (range 2.0–15.4), and the level was always over the normal range. All patients' temporal artery biopsies were positive.

**Conclusion:**

Alongside PAMM lesions, cotton wool spots, anterior ischemic optic neuropathy, and central retinal artery occlusion, choroidal ischemia is a key angiographic indicator in the diagnosis of GCA. It may be crucial to recognize these typical ischemic chorioretinal signs while diagnosing GCA.

## Introduction

Giant cell arteritis (GCA) is a medium to large vessel granulomatous vasculitis of autoimmune etiology with predilection to the cranial branches of the aortic artery [1–32] GCA has multisystem manifestations (new onset temporal headache, jaw claudication, low grade fever), propensity to the elderly population with an average age of onset of 75 years, and a strong female predominance [1, 2]. Since the involvement of the contralateral eye can increase to 60% when left untreated [8], visual loss is the most feared and irreversible complication of GCA, and therapy with a high-dose corticosteroid (and most recently tocilizumab) lowers the incidence of blindness. Vision loss results from either central retinal artery occlusion (CRAO) or posterior ciliary artery (PCA) occlusion manifesting as arteritic anterior ischemic optic neuropathy (A-AION) [10, 12]. The only way to diagnose many ischemic events occurring outside of the papillo-macular area is with intravenous fluorescein angiography, indocyanine angiography or optical coherence tomography angiography (OCTA). These events can involve the choroid (choroidal ischemia) [11, 12] or the retina (cotton-wool spots (CWS) and paracentral acute middle maculopathy (PAMM) [13–16]. The purpose of this study is to describe such circulatory ischemic events in a case series of GCA using multimodal imaging of the choroidal and retinal circulation.

## Methods

This multicenter, retrospective, observational case study examined the multimodal imaging results for 16 eyes of 8 patients treated for GCA at 6 ophthalmology clinics between January 2013 and December 2020. The descriptive study received ethical committee approval, the researchers agreed to a confidentiality agreement, and informed consent was not required. Data were collected from comorbidities, constitutional symptoms, acute phase reactant tests (erythrocyte sedimentation rate (Westergren) (ESR) and C-reactive protein (CRP), initial and final best corrected visual acuity.

The American College of Rheumatology categorization criteria, which call for three or more of the following signs or symptoms, were used to diagnose GCA: (1) age  ≥ 50 years; (2) headache of new onset; (3) temporal artery tenderness to palpation and decreased pulsation; (4) ESR  ≥ 50 mm/hr; and (5) temporal artery biopsy showing a predominance of mononuclear cell infiltration or granulomatous inflammation of the vessel wall, usually but not necessarily accompanied by multinucleated giant cells. An elevated level for CRP is set as a value above 1.0 mg/dL.

At presentation, patients underwent fundus photography, near-infrared reflectance (Spectralis, Heidelberg Engineering, Heidelberg, Germany), fluorescein angiography (Carl Zeiss Meditec or Topcon Medical Systems), spectral domain (SD) OCT (Spectralis, Heidelberg Engineering), or swept-source OCT and OCT angiography (OCT-A) (Topcon Medical Systems, Oakland, New Jersey, USA). The digital images of each patient were grouped for a multimodal study.

## Results

Eight patients (16 eyes) were identified and collected for the study, 6 were women (75%) and the age ranged from 68 to 83 years (mean 74.5 years). All patients reported constitutional symptoms (headache, scalp tenderness, neck pain, fever, weight loss, malaise, fatigue, jaw claudication) (Table 1). ESR and CPR values showed moderate elevation in most cases. Ocular ischemic damage was noted to be bilateral in 5 patients (62.5%). Choroidal ischemia was documented in 12 eyes (75%), PAMM, CWS and A-AION in 8 eyes each (50%), and CRAO in one eye (6%) (Table 1). Mean initial best spectacle corrected visual acuity (VA), after conversion to Log MAR, was 20/47 OD (oculus dexter) and 20/115 OS (oculus sinister) (range 20/20 to CF both eyes) while mean final visual acuity was 20/27 OD and 20/94 OS (range 20/20 to 20/80 OD; 20/20 to 20/800 OS) with a mean follow-up of 25.5 months (range 3–60).

**Table 1:** Demographics, clinical complaints, initial and final visual acuity and multimodal analysis findings in 8 patients with GCA

Case	Sex	Age	Visual Com- plaint	Syste- mic Signs	Initial VA OD	Initial VA OS	Final VA OD	Final VA OS	FU	ESR/CRP	Choroi- dal Ischemia	CRAO	PAMM	CWS	A-AION
1	F	70	Visual loss OS	Yes	20/20	CF	20/20	20/200	48	99/6.3	OU	OD	OD	OU	OD
2	F	78	Visual loss OS	Yes	CF	20/80	20/60	20/25	60	74/2.5	OU	No	OU	OU	OU
3	F	75	Visual	Yes	20/50	20/30	20/25	20/20	12	60/10.4	OU	No	OU	OS	OD
			loss OS												later
4	F	68	Visual loss OS	Yes	20/70	20/20	20/25	20/20	36	110/7.5	OD	No	OU	No	OD
5	F	72	Visual loss OS	Yes	20/20	20/800	20/20	20/200	36	4/2.0	OU	No	No	OU	No
6	F	79	Visual loss OS	Yes	20/30	CF	20/30	20/500	3	92/15.4	OS	No	OS	OS	OS
7	M	71	Visual loss OS	Yes	20/20	20/20	20/20	20/800	3	59/2.9	OS	No	No	No	OS
8	M	83	Visual loss OS	Yes	20/25	20/40	20/25	20/40	6	48/2.8	OS	No	No	No	OS

*F* female, *M* male, *OD* oculus dexter, *OS* oculus sinister, *OU* oculus uterque, *VA* best spectacle corrected visual acuity, *CF* counter fingers, *ESR* erythrocyte sedimentation rate (mm/hr), *CRP* C-reactive protein (mg/dL), *PAMM* paracentral acute middle maculopathy, *CWS* cotton-wool spots, *A-AION* arteritic anterior ischemic optic neuropathy, *CRAO* central retinal artery occlusion, *FU* follow up (months)

## Case reports

### Case 1

**Fig.1:**
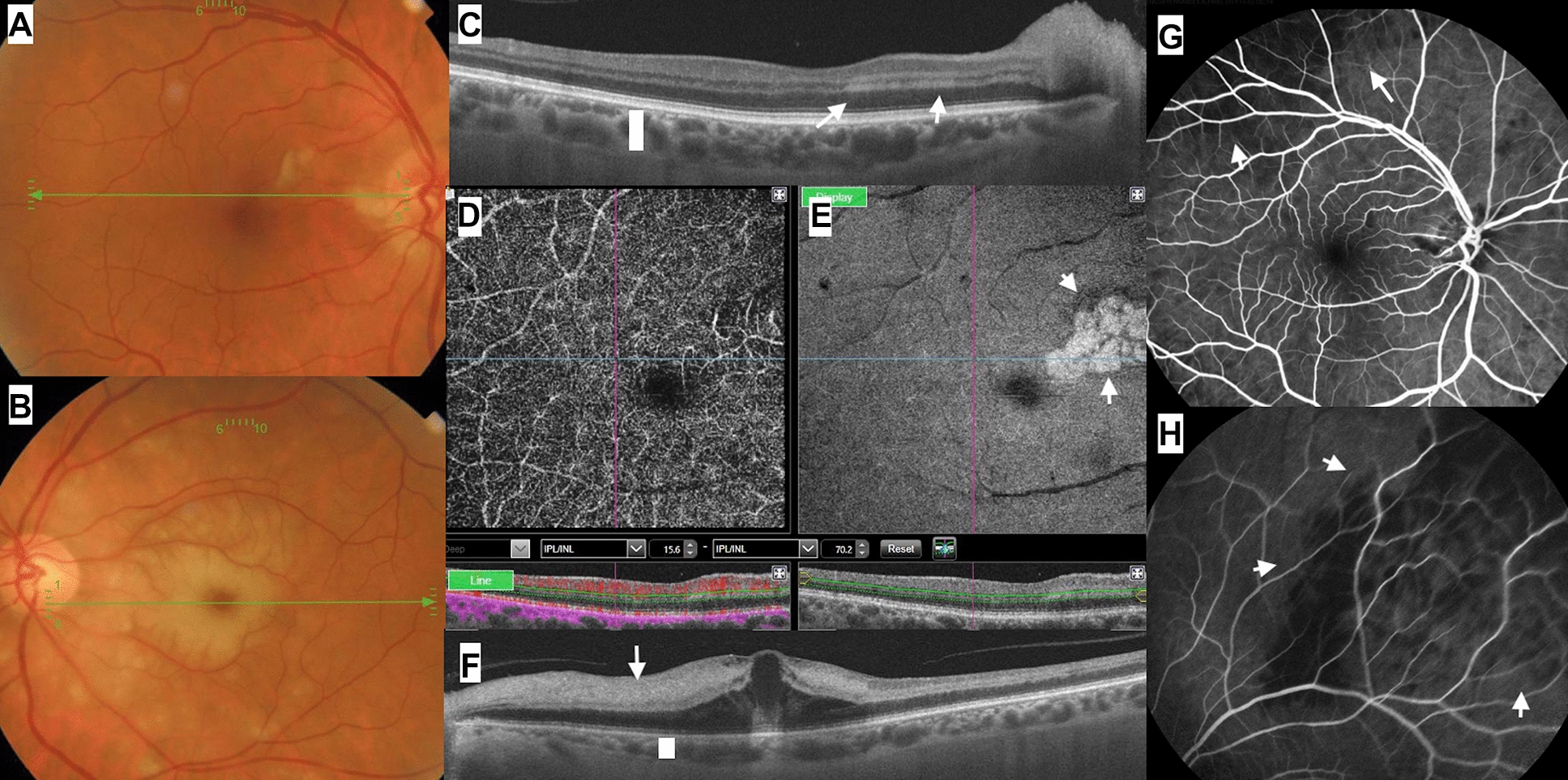
(Case 1). Posterior pole reveal bilateral cotton-wool spots and and a cherry red spot in the left eye (**A**, **B**). PAMM lesions are noted in the right macula on OCT (**C**) and more so on OCTA (**D**, **E**). OCT of the left macula (**F**) demonstrates opacification of the retinal layers and severe swelling of the fovea. On fluorescein angiography, superior temporal choroidal hypoperfusion assuming a triangular shape is noted bilaterally (**G**, **H**)

A 70 years-old Caucasian woman presented with visual loss in the OS of 5 days duration. She had pain in the left temple, mandible and neck, loss of strength in the left arm, loss of weight and loss of appetite. Visual acuity (VA) was 20/20 OD and 20/1200 OS, with a left afferent pupillary defect. Fundoscopy OD revealed blurry disc margins and cotton-wool spots while OS had a cherry red spot from CRAO (Fig. 1). Macular OCT scans allowed visualization of a hyperreflective band at the level of the inner nuclear layer (INL) sparing the outer nuclear layer and characteristic of paracentral acute middle maculopathy (PAMM) lesions (Fig. 1). OS showed total hyperreflective band with marked macular edema. OCT-A allowed a superior visualization of the ischemia of the deeper capillary plexus (Fig. 1e). Choroidal ischemia was evident angiographically in OU (oculus ulterque). Pain was reduced by corticosteroid pulse treatment, and VA improvement to 20/200 OS.

### Case 2

**Fig.2:**
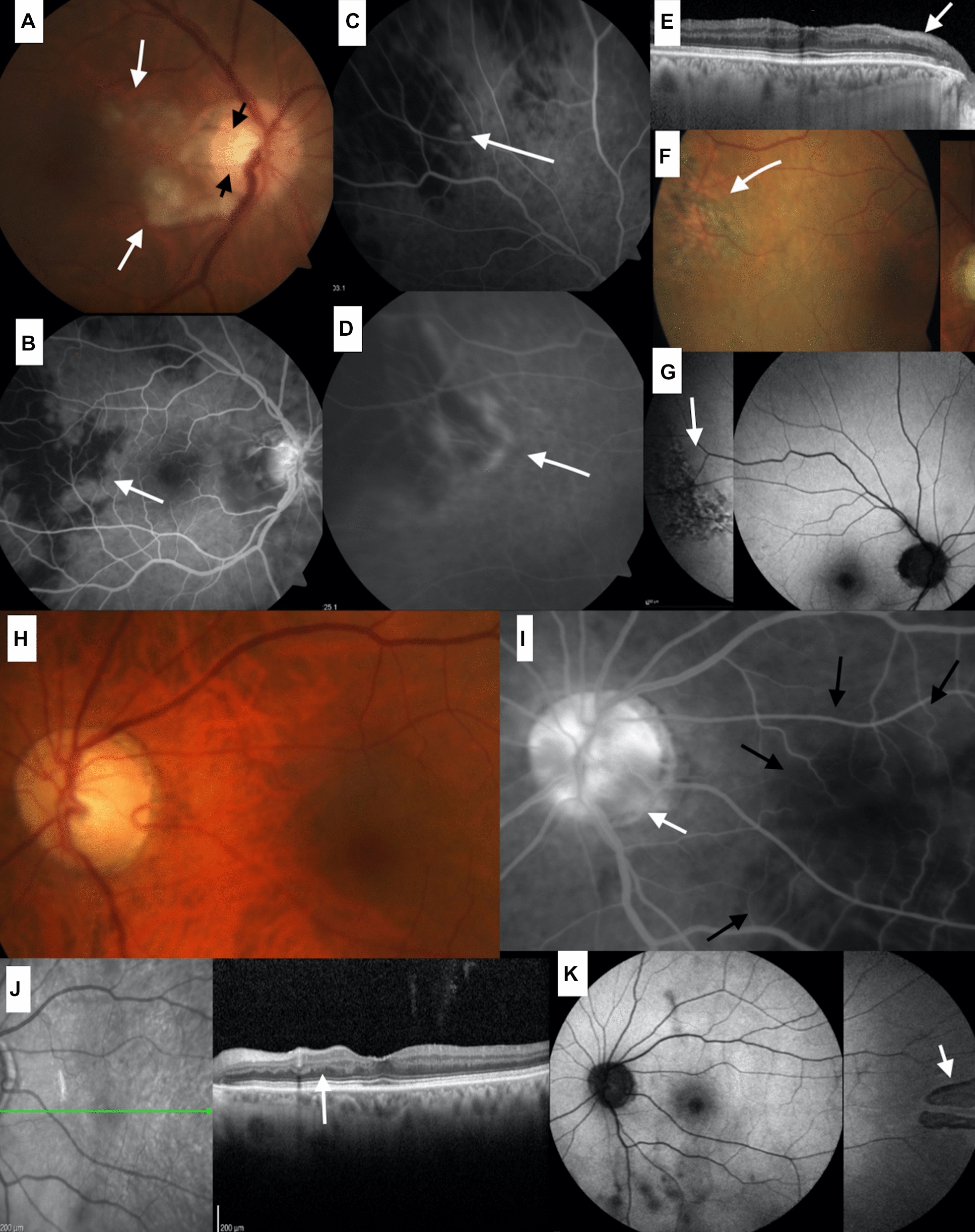
(Case 2). Fundus photographs of the posterior pole of the right eye shows occlusion of the 2 cilioretinal arteries (arrows- **A**). Areas of choroidal hypoperfusion temporal to fovea (arrow- **B**), along the superotemporal arcade (arrow- **C**) and temporal midperiphery (arrow- **D**) characteristic of the Amalric sign. OCT shows atrophy of inner retina in the area of the cilioretinal occlusion of the right eye (arrow-**E**). Amalric sign is again noted on color fundus of the right midperiphery (arrow- **F**) and on the corresponding aufluorescent image (arrow- **G**). Color photograph of the left posterior pole (**H**). Fluorescein angiography in its late transits demonstrate bilateral choroidal ischemia involving the fovea (black arrows- **I**) as well as sectorial hypoperfusion of the optic disc nasally and temporally (**I**- white arrow). Foveal OCT displays the irregular thickness of the inner nuclear layer of the eye (arrow- **J**). Amalric sign is noted on autofluorescent image of the temporal midperiphery of the left eye (arrow- **K**)

A Caucasian woman in her 78 s presented with a sudden loss of eyesight OU. She had recently had general malaise, severe weight loss, and jaw claudication, with the dentist attributing her jaw pain to a root-canal issue. No prior history of headaches existed. VA was 20/800 OD and 20/80 OS with a left afferent pupillary defect. Fundus examination revealed CWS OU (Fig. 2). OCT of the macula revealed hyperreflective lesions at the level of the INL OU (Fig. 2). Choroidal ischemia and optic disc leakage were evident OU on fluorescein angiography (FA) (Fig. 2). Her general condition significantly improved when she was promptly started on 60 mg of prednisone taken orally every day. After 2 weeks, visual acuity improved to 20/70 OD and 20/25 OS with partial resolution of CWS.

### Case 3

**Fig.3:**
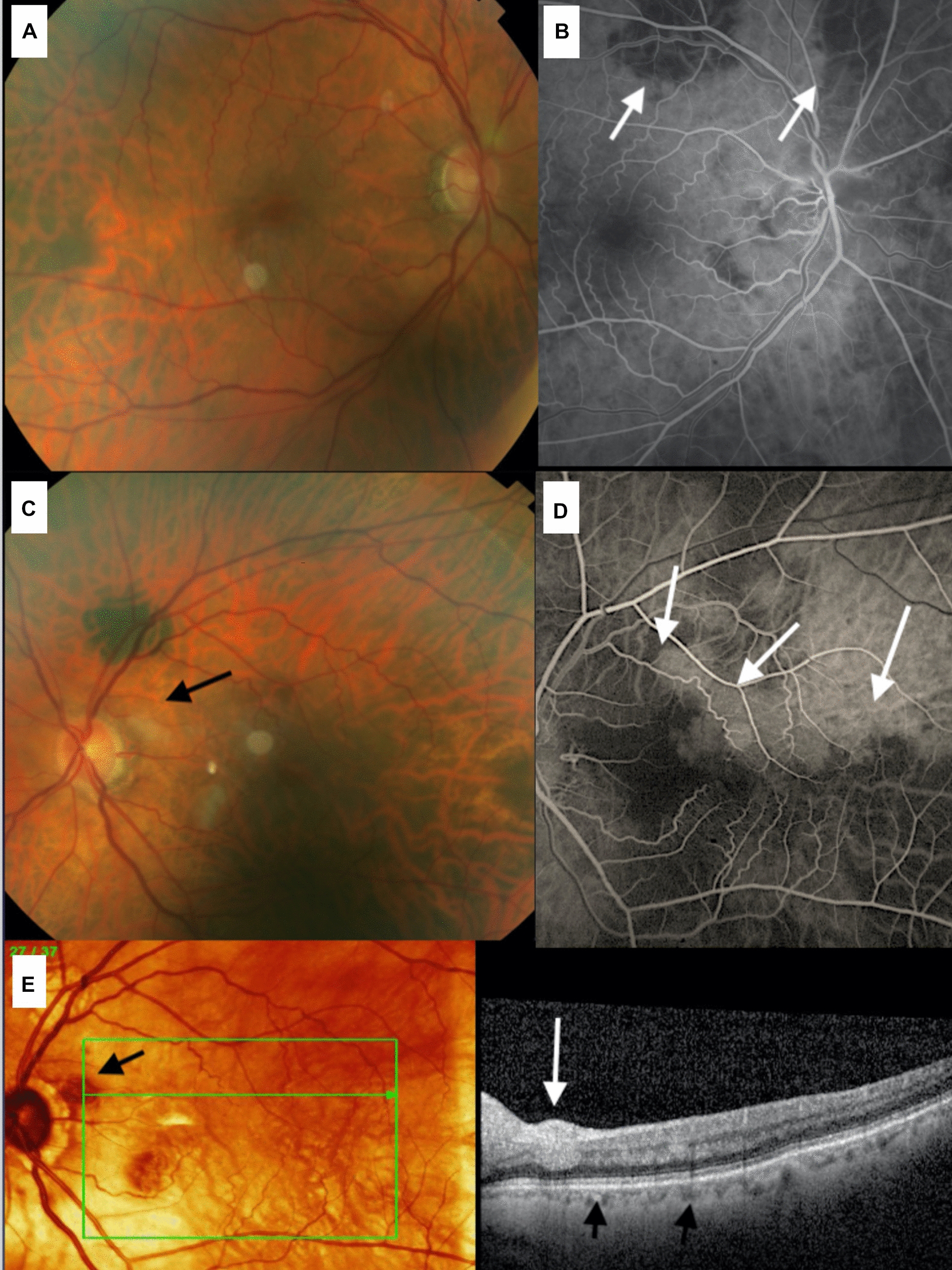
(Case 3). The right fundus (**A**) shows areas of severe delay in choroidal perfusion (**B**—arrows). The fundus of the left eye shows a cotton wool spot (black arrow -**C**). There is an inferotemporal sector of choroidal hypoperfusion (arrows- **D**) in the left eye. A cotton wool spot is noted temporal to the disc in the left eye (arrows 3E)

A 75 years-old Caucasian man presented with moderate visual loss OD of few days with visual acuity of 20/50 OD and 20/30 OS, accompanied by temporal headache. Multimodal imaging revealed CWS, PAMM lesions (OCT) and choroidal ischemia (FA) OU (Fig. 3). One week after oral prednisone (60 mg), VA improved to 20/25 OD.

### Case 4

**Fig.4:**
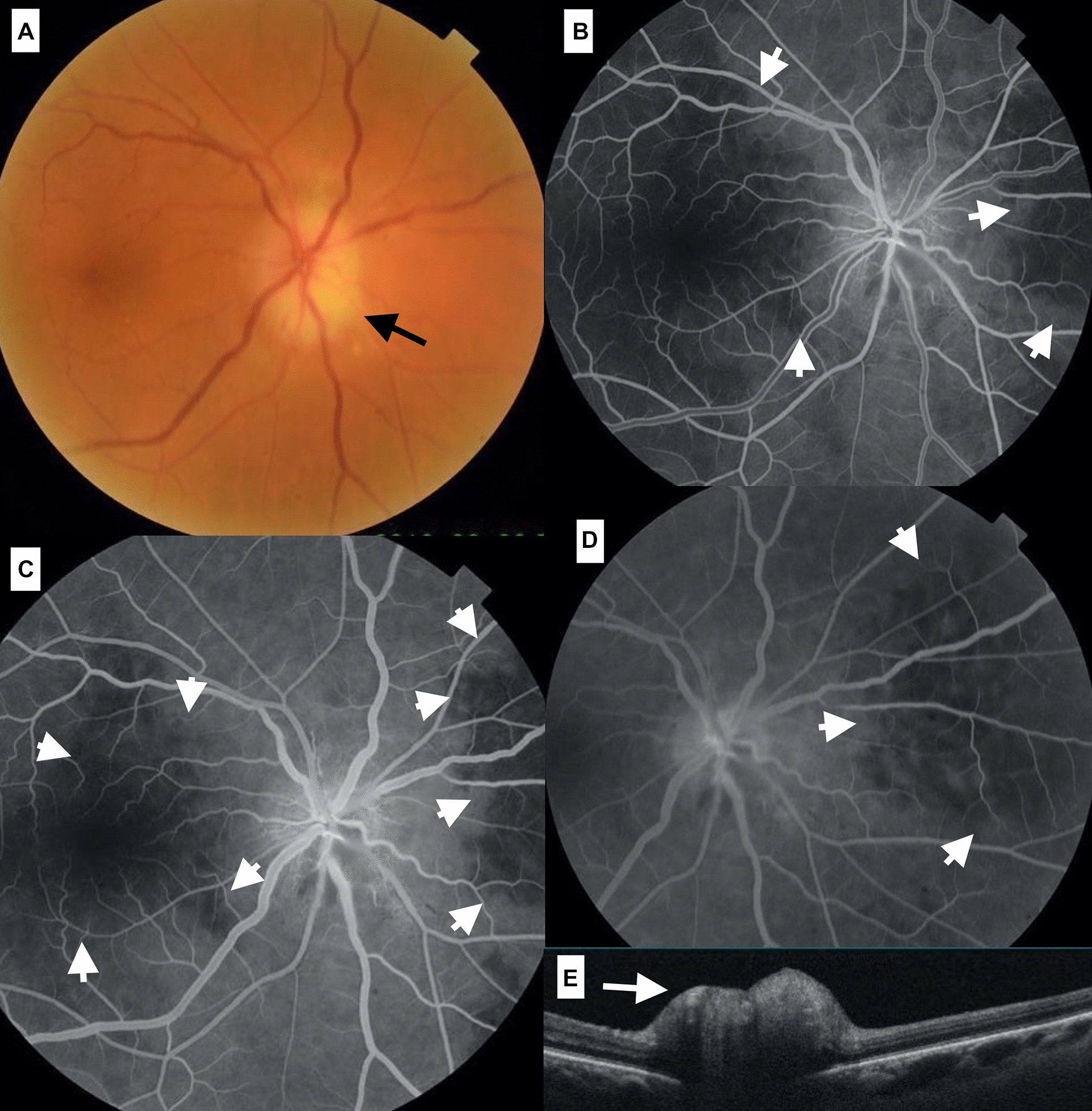
(Case 4). Pale disc swelling of the optic disc is noted in the right fundus (**A**). Angiography of the right eye revealed nasal sectorial choroidal ischemia (**B**, **C**, **D**) with disc edema and normal macular perfusion. OCT confirms the disc elevation (**E**)

A 68 years-old female patient presented to the emergency room for increasing frontal headache of 20 days duration and was reassured following a normal brain MRI. She subsequently noted mandibular pain and VA drop to 20/70 in OD and 20/25 in OS. Moderate edema of the optic disc was noted in OD. FA revealed nasal sectorial choroidal ischemia, disc edema and intact macular perfusion (Fig. 4). With cleared disc edema, pulse corticosteroid therapy improved VA to 20/30 OD in 48 h and 20/25 OD in 11 days.

### Case 5

A 72 years-old Caucasian woman complained of sudden visual loss of the OS of 3 days duration preceded by headache of 3 months duration. VA was 20/20 in the OD and 20/800 in the OS. Fundus examination showed normal-looking optic discs and CWS in OU, more intense in OS with some whitening of the retina in the posterior pole. PAMM lesions were visible OU on OCT. Choroidal ischemia was noted angiographically OS.

ESR was normal (4 mm/hr) but a CRP was elevated (2.0 mg/dL). An increase in the signal intensity around the left temporal artery was noted on brain MRA. Temporal artery biopsy was positive for GCA. Intravenous pulse therapy with 1 g of methylprednisolone for 5 days was followed by oral prednisone and methotrexate and by VA improving to 20/200 OS.

### Case 6

**Fig.5:**
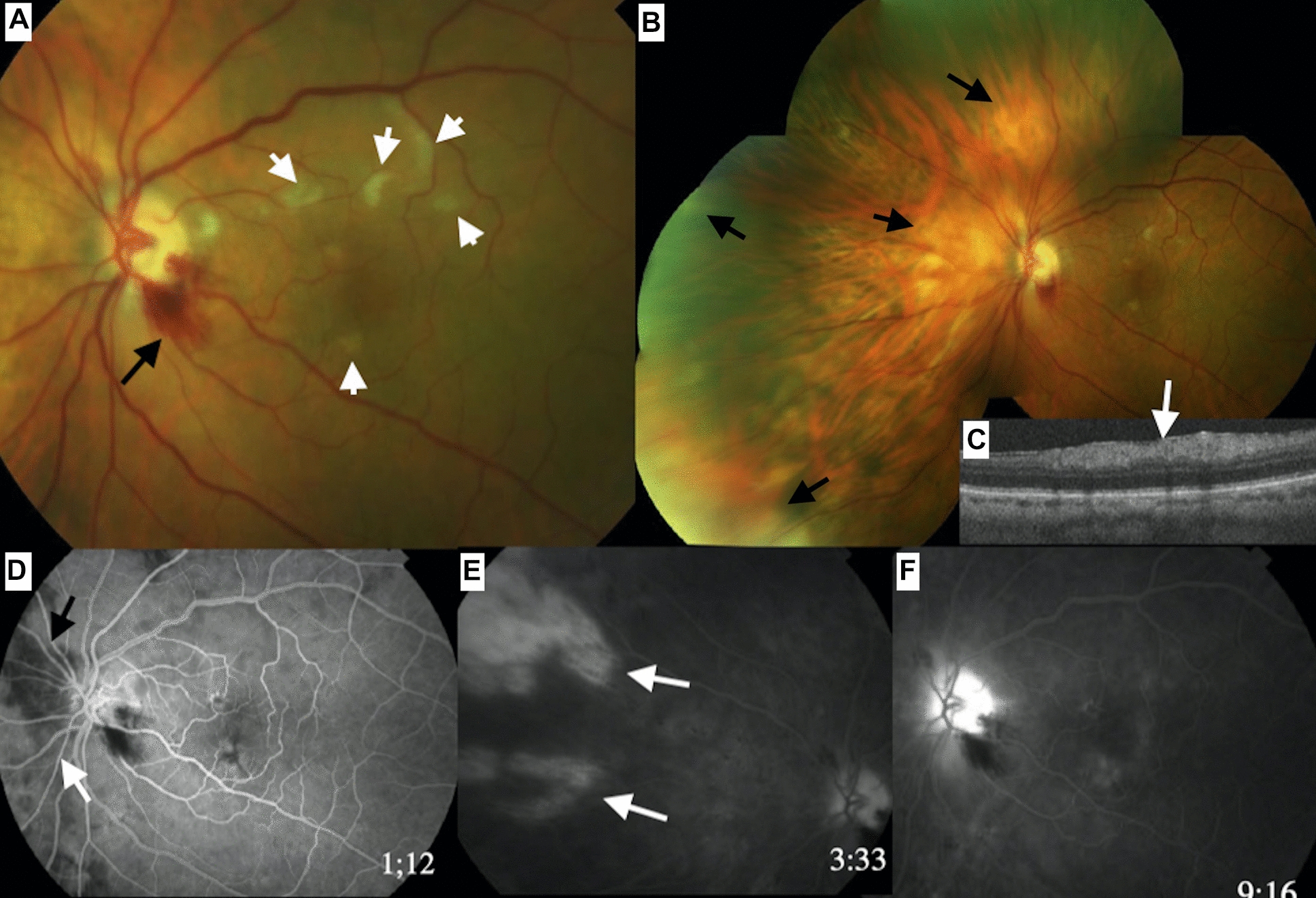
(Case 6). Multiple cotton-wool spots (white arrows- **A**, **B**, **C**) are present in the posterior pole of the left eye. Peripapillary nerve fiber hemorrhages (black arrows- **A**, **B**) are noted. Hypopigmented triangular area nasal to the disc (**A**, **B**, **D**) corresponds to an area of choroidal hypoperfusion. Amalric sign is present with typical triangular areas of hyperfluorescence in temporal midperiphery (**E**). Disc staining is noted in the late phases of fluoprescein angiography (**F**)

A 79 years-old Caucasian woman complained of visual loss in OU. VA was 20/30 OD and counting fingers OS (Fig. 5). After obtaining a temporal artery biopsy (positive for GCA), corticosteroid treatment allowed partial improvement of the VA to 20/500.

### Case 7

A 71 years-old Caucasian man complained of visual loss OS. VA was 20/20 OD and 20/800 OS. The fundus examination revealed optic discs that were normal. FA revealed hyperfluorescent optic disc and choroidal ischemia OS. VA did not improve, presumably as a result of the delayed start of corticosteroid therapy.

### Case 8

An 83 years-old Caucasian man with polymyalgia rheumatica complained of visual loss OS to 20/60. He had a left afferent pupillary defect, optic nerve head swelling, hyperfluorescent optic disc, and angiographic choroidal ischemia. VA OS improved to 20/40 after prompt administration of corticosteroid. The patient had also polymyalgia rheumatica.

## Discussion

Compared to other ischemic signs such as PAMM, CWS, or A-AION, choroidal ischemia was seen more frequently in the current case series. The impairment of vascular supply was detected even with a preserved VA. All patients (100%) had VA loss in at least one eye; choroidal infarction was present in 12 eyes (75%); PAMM lesions were present in 8 eyes (50%); CWS was present in 8 eyes (50%); A-AION was present in 8 eyes (50%); and CRAO was present in one eye (6.2%). The understanding of the posterior vascular circulation has improved as a result of this multimodal retinal examination.

In vivo FA studies [10] have shown that the posterior ciliary artery (PCA) supply the choroid (Table 2) in a strictly segmental flow, hence the choroidal vascular bed has watershed zones situated between the various PCAs and the short PCAs. The location of multiple water shed zones of the short PCA in the macular choroid makes the macular choroid more vulnerable to ischemic disorders than other parts of the choroid. Ischemic events in the long PCA impact the choriocapillaris circulation in the periphery and may also be asymptomatic. The recognition of such ischemic damage justifies prompt corticosteroid treatment to prevent further visual damage; in this case series, all patients had choroidal ischemia which may represent a cardinal sign of GCA.

**Table 2:** Ischemic findings per site and rate in previous literature and updated values

Ocular ischemia manifestations	TEST	Reported rate % (literature review before 2016)18	Updated rate%	Author	Publication year	Vascular supply
Optic nerve ischemia						
A-AION	Funduscopy; FA	88–92.3 [18]	41% (69/170) [31] 33% (22/66) [30] 7% (17/245) [32] 12% (18/146) [32]	Hayreh [31] Glutz von Blotzheim [30] Vodopivec [32]	2021 1997 2018	circle of ZinnHaller (short branches PCA)
Retinal ischemia						
CRAO	Funduscopy; FA	4–14.1 [18]	12% (20/170) [31] 15% (7/47) [30]	Hayreh [31] Glutz von Blotzheim [30]	2021 1997	Central retinal artery
CWS	Funduscopy	up to 33 [18]				Superficial vascular plexus
PAMM	OCT; OCT-A	0	17% (16/96)	Mairot [19]	2021	Middle capillary plexus
Choroidal ischemia	FA; ICG; SSOCTA	rare	36% (17/47)	Glutz von Blotzheim [30]	1997	long branches PCA
Anterior segment ischemia	Slit lamp exam	very rare	Rare	Tran^23^	2018	anterior ciliary, arteries, long PCA, anatosmotic connections from anterior choroid

*PCA* posterior ciliary arteries, *A-AION* arteritic anterior ischemic optic neuropathy, *CRAO* central retinal artery occlusion, *CWS* cotton-wool spot, *PAMM* paracentral acute middle maculopathy, *FA* fluorescein angiography, *ICG* indocyanine green angiography

Unrecognized by routine funduscopy, asymptomatic microvascular occlusions in the choroid and retina were discovered using multimodal imaging analysis. There may be more instances of choroidal ischemia with no filling in the periphery than previously believed due to the involvement of the long branches of the PCA (Table 2). Nemiroff et al. [24] noted that swept source OCT-A did provide a simple noninvasive tool to evaluate choroidal ischemia in 3 cases with GCA. While choroidal ischemia is often asymptomatic, ensuing visual loss have been reported when involving the macula or the papillomacular bundle [25–29]. Despite the fact that choroidal ischemia is described as being rare in the present literature [18, 25–29], it is probable that this irregularity of the choroidal circulation is frequently overlooked. The current research and other communications [30, 31] confirm that choroidal ischemia is frequently seen. (Table 2). On one side, our report analyzed the choroidal hypoperfusion in a qualitative way using FA. On the other side, Pellegrini et al. [22] measured quantitatively the choroidal vascularity index in eyes with A-AION using OCT-A. There was a subtle (4–6%) decrease of the choroidal vascularity index in eyes with A-AION vs. eyes with non-A-AION or vs. control eyes. The most recently characterized instance of vascular involvement is PAMM [33]. This hyperreflective parafoveal white band is located at the level of INL, is best seen on OCT-A, and develops into deeper layer atrophy as a result of selective obstruction of the deeper retinal vascular plexus [13]. A-AION, CWS, and CRAO are well recognized ischemic effects of GCA (Table 2).

In the literature, there is a significant disparity between the incidence of fundus abnormalities among GCA patients, which ranges from 12 to 70% [32]. For instance, it appears that the rate of A-AION is overestimated while the rate of PAMM is understated for several reasons: (1) referral bias in studies emanating from tertiary centers vs. population-based studies; (2) the prevalence of PAMM lesions is increasing as a result of the development of new technologies (OCT, OCT-A).

GCA is a systemic disease of variable presentation and duration. Marked elevation in acute phase reactants is one hallmark of GCA: 90% have ESR  > 50 mm/hr, 10% have ESR  < 50 mm/hr, and only 3.6% have ESR  < 30 mm/hr [17]. Temporal artery biopsy (granulomatous inflammation with giant cells and rupture of the internal elastic lamina) [3–6] remains the “gold-standard” diagnostic test with a sensitivity exceeding 90%. Ultrasound of the temporal arteries may substitute for the need of a biopsy in some cases. Including multimodal imaging can aid in the rapid identification of GCA even before the biopsy findings are known, facilitating early treatment.

Patients with suspected GCA are advised to undergo multimodal imaging as it may help in showing asymptomatic hypoperfusion areas evident findings of A-AION and CRAO. Multimodal imaging allowed the clinicians to view topographically the areas of ischemia in GCA. GCA is a systemic disease of very variable presentation and high suspicion index is required especially if all laboratory and pathology tests are negative or borderline inviting multimodal imaging of the retina and choroid to be the cardinal index in the diagnosis of GCA.

## Conclusions

The discovery of delayed sectorial choroidal filling on multimodal exams should raise the highest index of suspicion for GCA. Since it is a medical emergency and should lead to rapid investigation and aggressive treatment.

Choroidal hypoperfusion was present in all current cases of GCA and may represent a cardinal sign. Furthermore, the study reaffirms the importance of detecting PAMM lesions on multimodal retinal imaging, which is an important tool to use to study topographic circulatory disturbances in GCA.

## Data Availability

All the data supporting our findings are available through e-mail request from the corresponding author. The datasets generated during and/or analyzed during the current study are available from the corresponding author on reasonable request.

## Abbreviations

GCA: Giant cell arteritis
ESR: Erythrocyte sedimentation rate
CRP: C-reactive protein
A-AION: Arteritic anterior ischemic optic neuropathy
OCT-A: Optical coherence tomography-angiography
FA: Fluorescein angiography
PAMM: Paracentral acute middle maculopathy
OD: Oculus dexter
OS: Oculus sinister
OU: Oculus uterque
INL: Inner nuclear layer
CRAO: Central retinal artery occlusion
CWS: Cotton-wool spots
PCA: Posterior ciliary arteries
